# Efficient Degradation of Ciprofloxacin in Water over Copper-Loaded Biochar Using an Enhanced Non-Radical Pathway

**DOI:** 10.3390/molecules28248094

**Published:** 2023-12-14

**Authors:** Ting Guo, Qinyu Yang, Ruoqi Qiu, Jie Gao, Jingzhuan Shi, Xiaoyun Lei, Zuoping Zhao

**Affiliations:** School of Chemistry and Environmental Science, Shaanxi University of Technology, Hanzhong 723001, China15991023008@163.com (R.Q.); shijingzhuan0923@sina.com (J.S.);

**Keywords:** copper-loaded biochar, ciprofloxacin degradation, peroxymonosulfate activation, non-radical pathway

## Abstract

The development of an efficient catalyst with excellent performance using agricultural biomass waste as raw materials is highly desirable for practical water pollution control. Herein, nano-sized, metal-decorated biochar was successfully synthesized with in situ chemical deposition at room temperature. The optimized BC-Cu (1:4) composite exhibited excellent peroxymonosulfate (PMS) activation performance due to the enhanced non-radical pathway. The as-prepared BC-Cu (1:4) composite displays a superior 99.99% removal rate for ciprofloxacin degradation (initial concentration 20 mg·L^−1^) within 40 min. In addition, BC-Cu (1:4) has superior acid-base adaptability (3.98~11.95) and anti-anion interference ability. The trapping experiments and identification of reactive oxidative radicals confirmed the crucial role of enhanced singlet oxygen for ciprofloxacin degradation via a BC-Cu (1:4)/PMS system. This work provides a new idea for developing highly active, low-cost, non-radical catalysts for efficient antibiotic removal.

## 1. Introduction

Ciprofloxacin (CIP) is a broad-spectrum antibiotic commonly used to treat infectious diseases, but it may also have some polluting effects on the water environment [1]. CIP is usually discharged into the water environment through human and animal excreta and wastewater [2]. In water bodies, CIP can interact with bacteria, algae, and other organisms, potentially affecting aquatic ecosystems [3]. Although CIP has some biodegradability, it can be degraded slowly. Liu et al. [4] investigated the pollution of antibiotics in different aquatic environments in Beijing and found that sulfonamides or quinolones were mainly present in surface water and groundwater, within which the concentration of CIP was 8.480 mg/L. However, CIP that entered the aquatic environment might interact with bacteria in the water, potentially leading to mutations in bacterial resistance and causing the problem of antibiotic resistance. In addition, ciprofloxacin may also have toxic effects on aquatic organisms [5].

Microbial degradation of CIP is one viable method [6]. Some bacteria and fungi have the ability to degrade ciprofloxacin, and by increasing the presence of these degrading bacteria, the degradation of CIP in water bodies can be promoted [7]. The use of specific absorbents, such as activated carbon [8], ceramic membranes [9], and nanomaterials [10], can effectively remove ciprofloxacin from water. Although adsorption is an optional solution, thorough CIP removal in water is still difficult. In recent years, advanced oxidation processes (AOPs), such as ozone oxidation [11], photocatalytic oxidation [12], UV irradiation [13], and Fenton-like reaction [14], can degrade CIP with oxidation. The Fenton-like reaction uses hydroxyl radicals produced by hydrogen peroxide and iron ions to perform the oxidative degradation of ciprofloxacin. In recent years, researchers have also developed some improved Fenton-like processes, such as electrochemical Fenton [15] and photoelectrochemical Fenton [16], for improving the removal efficiency of ciprofloxacin.

Straw biochar is a renewable material obtained using pyrolysis of straw [17], which has a high specific surface area and porous structure, so it is widely used in adsorption to remove organic pollutants in water [18,19], including ciprofloxacin [20]. Straw biochar is usually obtained with high-temperature pyrolysis of straw under oxygen-free or low-oxygen conditions. In order to improve the adsorption efficiency of biochar on CIP, researchers have also carried out various modification techniques, such as pickling [21], atmosphere activation [22], and functional group introduction [23]. Studies have shown that straw biochar has good adsorption performance and can effectively remove ciprofloxacin in water. The main mechanisms for removing ciprofloxacin with straw biochar may include physical adsorption [24] and chemical adsorption [2]. However, the regeneration of biochar affects ciprofloxacin removal efficiency.

Sulfate-radical-based AOPs using biochar or metal–biochar composites are an effective pathway to remove CIP in water completely. Researchers prepared a metal-supported biochar catalyst by loading metal nanoparticles such as iron [25], cobalt [26], or manganese [27] onto biochar. These catalysts have high specific surface area and rich functional groups to improve the efficiency of persulfate activation and ciprofloxacin degradation. Iron oxide-loaded biochar is usually prepared using a precipitation method or impregnation method. Researchers prepared iron oxide-supported biochar, which has a large specific surface area and porous structure, providing rich sites for the adsorption of CIP. In addition, the presence of iron oxides can not only improve the adsorption capacity of biochar but also generate hydroxyl radicals under light, further promoting the degradation of CIP. Xiong et al. [28] designed single atoms Fe_3_C from Enteromorpha-derived biochar, explaining that the mechanism enhanced peroxymonosulfate adsorption and activation on Fe_3_C, producing an efficient property toward nitenpyram removal. Biochar loaded with cobalt oxide has more surface chelating sites and polarities, as well as rich functional components, and has a strong affinity for metal ions through ion exchange, complexation, and electrostatic interaction. Manganese oxide-supported biochar with a large specific surface area and porous structure provided rich sites for the adsorption of ciprofloxacin. Reactive oxygen species are produced under appropriate conditions, thereby enhancing the oxidative degradation of CIP with physical adsorption and chemical degradation.

**Figure 1:**
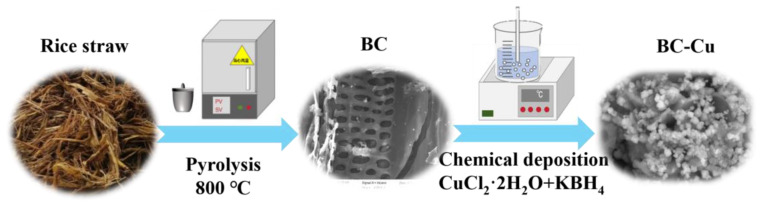
Synthesis scheme for rice straw-derived biochar and Cu-loaded biochar.

In this work, biochar loaded with metal oxides (Fe, Co, or Cu) was prepared and used for efficient PMS activation of CIP degradation (Figure 1). The batch experiment evaluated the performance of CIP removal via Fe-, Co-, and Cu-loaded biochar. Then, the ratio of copper content was further investigated. The factors affecting CIP removal were studied, verifying the ability of the materials in practice. In addition, reactive oxidative species pathways, including free radicals and singlet oxygen, were explained in detail. This work proposed a feasible way toward the synergistic mechanism of metal and metal–biochar compounds as well as CIP removal.

## 2. Results and Discussion

### 2.1. Catalyst Characterization

**Figure 2:**
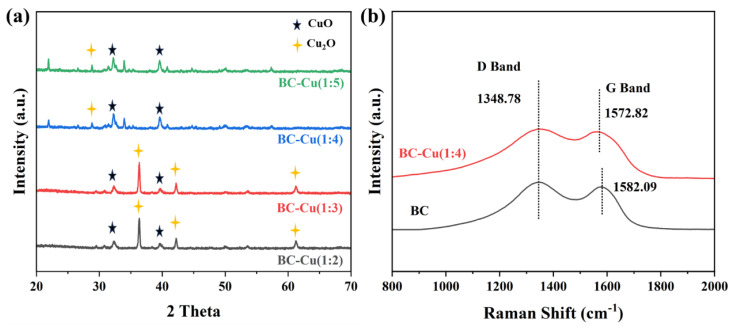
XRD patterns of BC-Cu series catalysts (**a**). Raman spectra of BC and BC-Cu (1:4) (**b**).

The XRD pattern was collected to confirm the phase and crystal structure of the prepared BC and BC-M materials. Figure S1 shows that the peak at 21.12° of the BC pattern is in agreement with the standard diffraction peak of derived biochar in previous publications [29]. As for BC-Co and BC-Fe, in addition to the characteristic peaks of biochar, the new phase and structure of Co_3_O_4_ (JCPDs NO.09-0418) and Fe_2_O_3_ (JCPDs NO. 02-1165) can be observed in Figure S2. In Figure 2, the XRD patterns of the BC-Cu series composites exhibit the sharp diffraction of Cu_2_O (JCPDs NO.99-0041) and CuO (JCPDs NO. 48-1548) [30] which indicate the successful formation of copper oxide nanoparticles on BC.

The disorder and defects of prepared BC and BC-Cu (1:4) were further studied with Raman spectra in Figure 2b; the prepared BC and BC-Cu (1:4) all showed a typical D band (1348.78 cm^−1^), which is derived from disorder and structure in carbon materials. The G band, representing symmetry and order, also could be found in BC (1582.09 cm^−1^) and BC-Cu (1:4) (1572.82 cm^−1^), while the characterized G band in BC-Cu (1:4) shifted to lower wavenumbers, suggesting possible Cu atom doping in the BC structure. Meanwhile, their intensity ratios (I_D_/I_G_) could be estimated to be 1.06 and 1.02 for BC and BC-Cu (1:4), respectively, reflecting the decreased defect. In addition, weaker peaks after copper modification could be observed, which indicated a decreased concentration of biochar and increased content of copper oxides in the prepared BC-Cu (1:4) [31].

**Figure 3:**
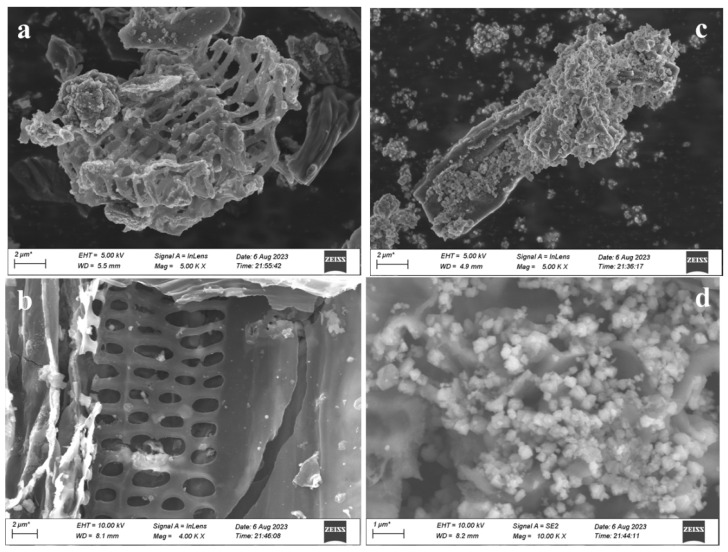
SEM images of BC (**a**,**b**) and BC-Cu (1:4) (**c**,**d**).

SEM images were used to study the morphology and surface variation after modification. From the SEM images of BC and BC-Cu (1:4) in Figure 3a,b, the rice straw-derived BC bulk showed a rough and irregular surface. The sponge-like structure exhibited a net-framework with visible 1~2 μm holes. After Cu modification (Figure 3c,d), the abundant copper oxide nanoparticles stacked onto the exterior surface and holes of the BC-Cu (1:4) catalyst. The particle size of the copper oxides ranged from 50 to 800 nm. The uniform distribution of copper oxide nanoparticles can be observed with SEM elemental mapping in Figure S3. The EDS spectrum and element content table of BC-Cu (1:4) showed that the content of C, O, and Cu was 36.05%, 40.75%, and 23.21%, respectively.

**Figure 4:**
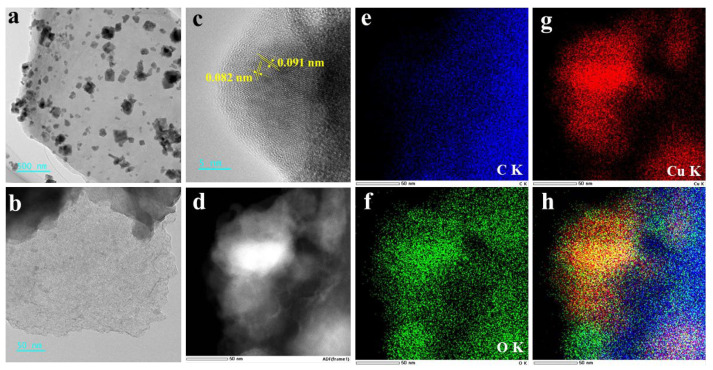
TEM image (**a**,**b**), HRTEM image (**c**), and HAADF image (**d**) of BC-Cu (1:4). Mapping images of element distribution in BC-Cu (1:4) (**e**–**h**).

TEM images were used to further specific investigation for BC-Cu (1:4). In Figure 4a, the copper oxides uniformly loaded onto the stacking of smooth carbon sheets, which pointed out the high percentage of copper oxides in BC-Cu (1:4). The thin sheets of BC had abundant micropores and mesopores as visible in Figure 4b. Furthermore, the lattice of 0.082 nm and 0.091 nm was indexed to that of CuO and Cu_2_O (Figure 4c), which corresponded to the phase and crystal structure in the XRD analysis results. The elemental mapping images in Figure 4d–h confirmed the existence of C, Cu and O. 

**Figure 5:**
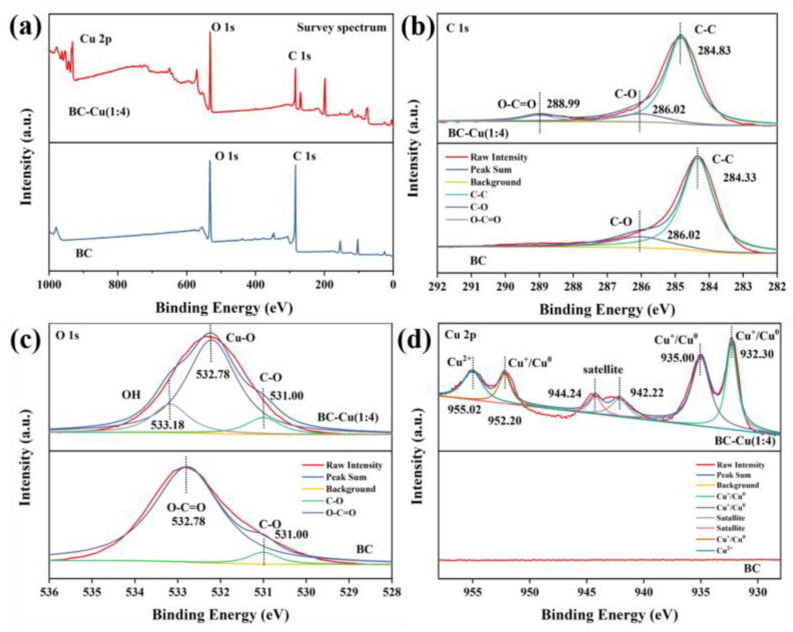
Survey XPS spectra (**a**) of BC and BC-Cu (1:4). HR-XPS spectra of C 1s (**b**), O 1s (**c**), and Cu 2p (**d**) in BC and BC-Cu (1:4).

The XPS analysis was provided to distinguish the chemical bonds and states of elements on the surface of BC and BC-Cu (1:4) [32]. As Figure 5a shows, Cu 2p of BC-Cu (1:4) could be observed after decoration. The high-resolution of C 1s, O 1s, and Cu 2p spectra in BC and BC-Cu (1:4) were analyzed as Figure 5b–d detail. There were two typical peaks of 284.33 eV and 286.02 eV in BC, which corresponded to the C-C and C-O, respectively. With the copper doping in BC-Cu (1:4), the peak 288.99 eV indexed to the O-C=O emerged. Meanwhile, the peak of the C-C shifted to a lower B.E., suggesting new bond generation. For Cu 2p in BC-Cu (1:4), the peaks of 932.30 eV and 952.20 eV confirmed the generation of Cu, Cu^+^, and Cu_2_O on the surface of BC-Cu (1:4). In addition, the peaks of 935.00 eV and 955.02 eV indicated the Cu^2+^ in CuO. Furthermore, there was a typical satellite peak of Cu 2p in BC-Cu (1:4) because of the high copper modification concentration.

### 2.2. Catalytic Performance Test

#### 2.2.1. Catalytic Performance of Samples

**Figure 6:**
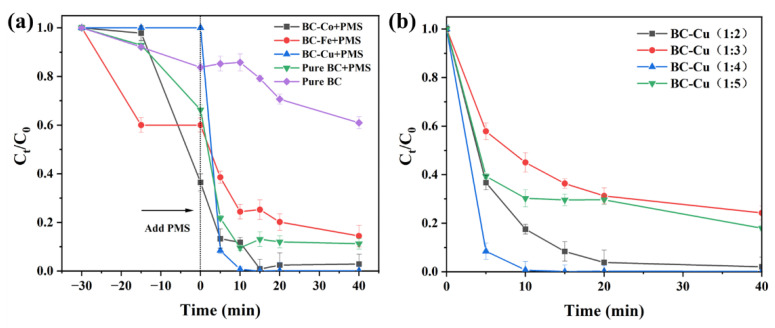
CIP degradation under different systems: metal-loaded BC (**a**) and BC-Cu with various Cu contents (**b**)**.** CIP concentration = 20 mg·L^−1^, initial pH = 6.80, catalyst dosage = 0.20 g·L^−1^, PMS concentration = 0.20 g·L^−1^.

In order to comprehensively evaluate the performance of BC and BC-M series materials in removing CIP, the adsorption and degradation of CIP for all materials, such as BC, BC-Fe, BC-Co, and BC-Cu(1:x), were compared. For the adsorption of CIP, as Figure 6a shows, without adding PMS in 30 min, the efficiency of removing CIP using BC, BC-Fe, or BC-Co was 17.03%, 40.49%, and 63.65%, respectively. However, it can be seen that BC-Cu barely removed CIP with adsorption, which is due to the copper loading hindering the pores and holes exhibited in the SEM images. For the PMS activation process, CIP removal with pure BC is 60.64% in 40 min, which is due to the adsorption of biochar; it is difficult to effectively remove CIP in water. However, when PMS was added into the BC/CIP system, 90% of CIP could be removed in 40 min. For other prepared catalysts BC-Fe, BC-Co, and BC-Cu, CIP removal was enhanced to 86.03%, 96.83%, and 99.99%, respectively. It is suggested that metal-loading onto BC can strengthen PMS activation. The ability of BC-M composites to activate PMS was much greater than that of BC, and the adsorption effect played only a secondary role; oxidative degradation was used as the dominant reaction to remove CIP. At the same time, the loading ratio of copper onto biochar was optimized. As Figure 6b shows, BC-Cu (1:2), BC-Cu (1:3), BC-Cu (1:4), and BC-Cu (1:5) could remove approximately 98%, 75%, 82%, and 99% of CIP, suggesting the optimal catalytic performance of BC-Cu (1:4). According to previous research, many nano-sized copper oxides are easy to aggregate, which could decrease the catalytic performance.

#### 2.2.2. Effects of Other Parameters on CIP Degradation

**Figure 7:**
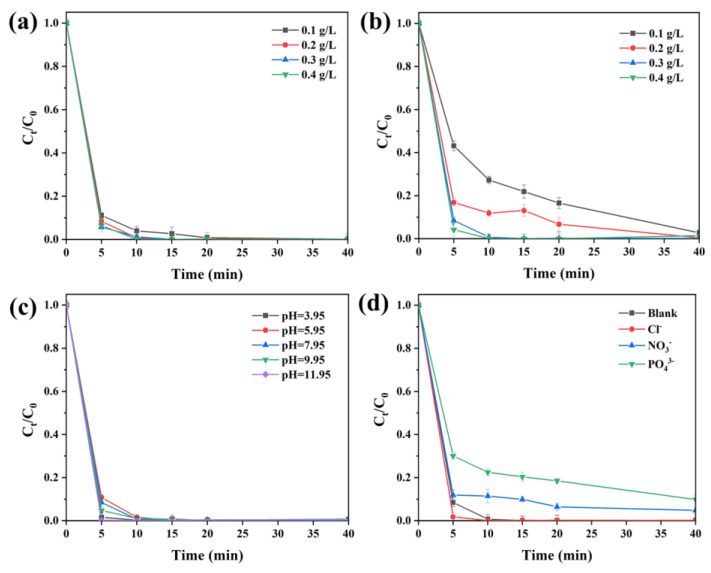
Influence of catalyst dosage (**a**), PMS concentration (**b**), pH value (**c**), and coexisting anions on CIP degradation (**d**).

Figure 7a shows the impact of different catalyst dosages (0.1 g/L, 0.2 g/L, 0.3 g/L, 0.4 g/L) on the degradation of CIP efficiency. It can be seen that for the system with the increased dosage, the removal efficiency of CIP increases insignificantly. As the amount of catalyst increased from 0.1 g/L to 0.4 g/L, the CIP removal rate increased and then decreased in 5 min, which was due to the dosage addition increasing the reaction site in the system [30]. However, the final removal rate with a different dosage achieved approximately 99.99% in 40 min, indicating that the increase in catalyst usage minorly affected the activation of PMS on CIP catalytic degradation. When the chemical agent is used (0.2 g/L), there should be more catalytically active sites in the reaction solution, resulting in superior activity. Therefore, 0.2 g/L of BC-Cu (1:4) was selected as the catalyst for the CIP degradation, and its catalytic properties were selected for further studies.

The effect of PMS concentration (0.1, 0.2, 0.3, 0.4 g/L) on CIP degradation was studied. The results are shown in Figure 7b. It can be seen that when the PMS concentration increased from 0.1 g/L to 0.4 g/L, the degradation efficiency of CIP increased from 56.43% to 97.08% within 5 min. The final 98%~99% occurred within 40 min. Considering the degradation and dosage, 0.3 g/L of PMS was chosen for further studies.

In general, the pH value of a reaction system affects the production of reactive oxidative species, so pH is one of the important influencing factors in redox systems [33]. Studies have shown that acidic conditions are more conducive to the activation of persulfate, while alkaline conditions are more favorable for inhibiting the production of SO_4_ [34]. Therefore, the effects of different pH conditions (3.95, 5.95, 7.95, 9.95, and 11.95) on the BC-Cu (1:4)-activated PMS degradation of CIP were systematically investigated. The results are shown in Figure 7c; as can be seen, the efficiency of CIP degradation at 40 min reached more than 99% at different pH levels (3.95, 5.95, 7.95, 9.95, and 11.95). Across these levels, in the first 5 min of the reaction, pH had a significant effect on the removal rate of CIP. The reaction rate increased from 90% to 96% with the pH increase from 5.95 to 9.95 in the first 5 min. At all levels, the CIP degradation reached ~99.99% at pH = 9.95 and 11.95 within 40 min. The results showed that under the conditions of strong acidity (3.95) and strong alkaline (11.95), it was more conducive to BC-Cu (1:4) activation PMS to generate active free radicals, thereby effectively removing the organic pollutant CIP. Under alkaline conditions, the Cu-OH complex on the surface of the material would increase the rate of free radical production. The pH condition experiment showed that the prepared BC-Cu (1:4) showed excellent degradation efficiency for CIP under wide pH conditions.

Inorganic anions such as Cl^−^, NO_3_^−^, and PO_4_^3−^ are widely existent in wild aquatic environments and could react with reactive species (SO_4_^−^ and •OH), affecting the catalytic reaction (Figure 7d). The results suggested that the addition of coexisting Cl^−^ and NO_3_^−^ was not conducive to the degradation of CIP because they would react with PMS to form fewer active substances (Cl_2_^−^ and NO_3_) [26]. Compared with other anions, CIP degradation was hindered about 10% in the presence of PO_4_^3−^, which could be due to PO_4_^3−^ having a strong affinity with copper oxides on the surface of BC via substituting O for the adsorptive HSO_5_^−^ complex [33].

**Figure 8:**
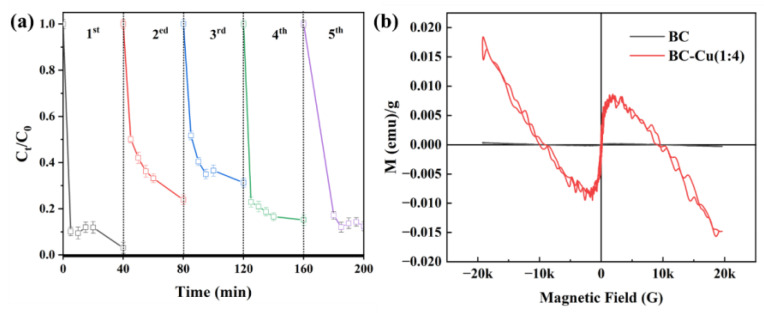
Cycle experiments of a BC-Cu/PMS/CIP system with conditions: CIP concentration = 20 mg·L^−1^, initial pH = 6.80, catalyst dosage = 0.2 g·L^−1^, PMS concentration = 0.3 g·L^−1^ (**a**); Vibrating sample magnetometer (VSM) of BC and BC-Cu (1:4) (**b**).

The stability of the material is one of the important indicators of performance in catalytic reactions. To evaluate the stability and reusability of the synthesized BC-Cu (1:4) composite, PMS was activated for CIP degradation. Under the conditions of the original pH, the PMS concentration of 0.2 g/L, and 0.2 g/L of BC-Cu (1:4), the cycle CIP removal rate with BC-Cu (1:4) is shown in Figure 8a. After 5 reactions, BC-Cu (1:4) still showed good catalytic degradation activity (>90%). The magnetic properties of the prepared catalytic materials used were investigated [35], and the results appear in Figure 8b; the M-H curve shows that the magnetization moments of the BC and BC-Cu (1:4) composites were about 0 emu/g and 0.008 emu/g, respectively. The M-H curve shows that the coercivity of the BC-Cu (1:4) composites was 0 through the origin, which proves that the catalyst was superparamagnetic. Compared with BC, BC-Cu (1:4) could be easily attracted using external magnetic field magnets, proving that the prepared composites had excellent magnetic separation performance, which is of great significance for the recovery and recycling of catalytic materials.

#### 2.2.3. The Possible Mechanism of CIP Degradation

**Figure 9:**
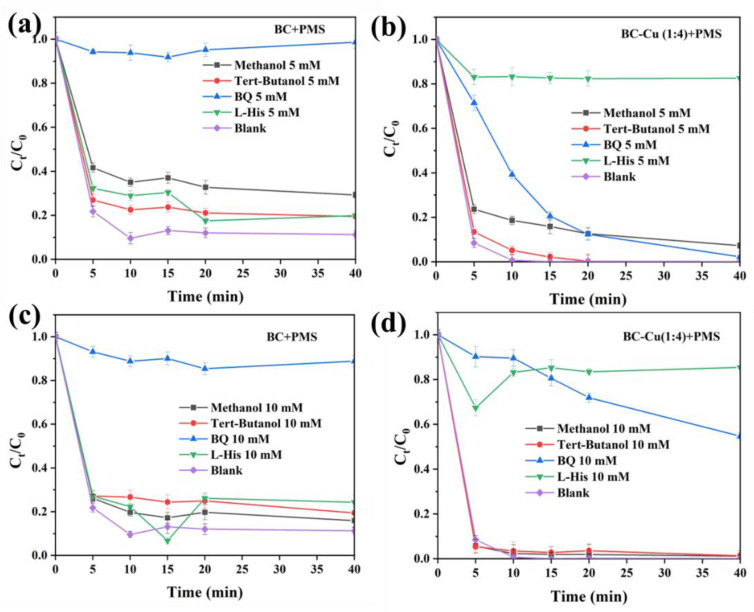
The inhibition of CIP degradation using trapping experiments with different agents and concentrations in BC/PMS (**a**,**c**) and BC-Cu (1:4)/PMS (**b**,**d**) systems. CIP concentration = 20 mg·L^−1^, initial pH = 6.80, catalyst dosage = 0.2 g·L^−1^, PMS concentration = 0.3 g·L^−1^.

Experiments trapping ROS in CIP degradation using different quenching agents in different concentrations in BC-Cu/PMS/CIP and BC/PMS/CIP systems were conducted to evaluate the roles of SO_4_^•−^, •OH, O_2_**·**^−^, and ^1^O_2_ [36,37]. Methanol (MeOH), tert-Butanol (TBA), and benzoquinone (BQ) were selected as scavengers of radical species in order to investigate the contributions of SO_4_^•−^, •OH, and O_2_**·**^−^, while L-Histidine was applied as the unique quenching agent for single oxygen (^1^O_2_). The CIP degradation under different trapping agents in Figure 9a–d indicates the significant participations of ROS, including radicals (SO_4_^•−^, •OH, O_2_**·**^−^) and a non-radical pathway (^1^O_2_) to CIP degradation, which corresponds with the results of the ESR analysis. Different degrees of inhibition toward CIP removal in different systems using methanol MeOH, TBA, BQ, and L-Histidine were investigated. As Figure 9a,c show, O_2_**·**^−^ played the key role toward CIP degradation in the BC/PMS/CIP system. However, greater inhibition was obtained with 5 mM L-Histidine in the BC-Cu (1:4)/PMS/CIP system than was obtained with other trapping agents, suggesting more generation of ^1^O_2_ in the BC-Cu (1:4)/PMS/CIP system. Otherwise, when the concentrations of BQ and L-Histidine increased to 10 mM, the inhibition of CIP degradation was increased. Only 46% and 14% of CIP was degraded in the BC-Cu (1:4)/PMS/CIP system with 10 mM BQ and L-Histidine, which further confirmed the enhanced catalytic property of Cu-decorated biochar for PMS activation.

**Figure 10:**
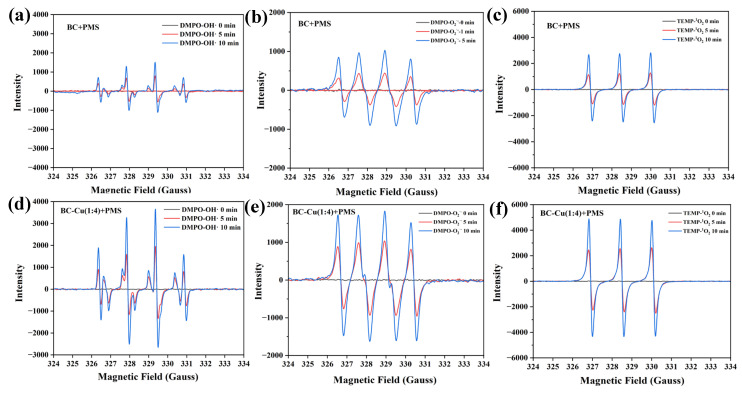
ESR signals of DMPO-•OH, DMPO-SO_4_^−^ (**a**), DMPO-O_2_**·**^−^ (**b**), and TEMP-^1^O_2_ (**c**) generating in BC/PMS (**a**–**c**) and BC-Cu (1:4)/PMS (**d**–**f**) systems. CIP concentration = 20 mg·L^−1^, initial pH = 6.80, catalyst dosage = 0.2 g·L^−1^, PMS concentration = 0.3 g·L^−1^.

The catalytic mechanism of PMS activation for the degradation of CIP was explored. EPR experiments detected the radicals generated in the reaction (Figure 10a,d). By employing 5, 5-dimethyl-1-pyrroline N-oxide (DMPO) as a trapping agent, the signals of DMPO-SO_4_^•−^ and DMPO-•OH appeared in both the BC-Cu/PMS/CIP and BC/PMS/CIP systems as expected in traditional sulfate-based AOPs [38]. Meanwhile, the signals of DMPO-O_2_**·**^−^ were also observed in different systems (Figure 10b,e), suggesting the participation of O_2_**·**^−^ in the oxidation process. In addition, 2,2,6,6-tetramethylpiperidine (TEMP) was applied as the spin trap to identify single oxygen (^1^O_2_) in the different systems. It can be observed in Figure 10c,f that obvious triplet-peak signals of TEMP-^1^O_2_ were detected in BC-Cu/PMS/CIP and BC/PMS/CIP systems, indicating the presence of ^1^O_2_ [39]. Notably, the intensity of the characteristic signals of DMPO-SO_4_^•−^, DMPO-•OH, DMPO-O_2_**·**^−^, and TEMP-^1^O_2_ triggered by the BC-Cu/PMS/CIP system are stronger than those triggered by the BC/PMS/CIP system, which indicated that the boosting generation of free radicals and single oxygen would be boosted due to the copper loading on biochar [40].

Based on the above analyses, the possible mechanism of CIP degradation using enhanced PMS activation over copper oxide-decorated biochar is proposed and illustrated in expressions 1–7. First, HSO_5_^−^ in a reaction system is directly activated to produce SO_4_^•−^ with Cu(II) and Cu(I) (Equations (1)–(3)). Meanwhile, SO_4_^•−^ will react with OH^−^ in the system to generate another free radical, •OH (Equation (4)). On the other hand, the production of **·**O_2_^−^, involved in CIP degradation, is related to the reaction of ≡Cu(II)-OH, HSO_5_^−^ and H_2_O. Then, the O_2_^·−^ reacts with •OH to ^1^O_2_. The attack of SO_4_^•−^, •OH, O_2_**·**^−^, and ^1^O_2_-oxidized CIP was on smaller molecular, even direct, mineralization.

≡Cu(II)-OH + HSO_5_^−^ → ≡Cu(I)-(OH) + SO_5_^−^ + H^+^ (1)

≡Cu(I)-OH + HSO_5_^−^ → ≡Cu(I)-OH-OSO_3_^−^ + OH^−^ (2)

≡Cu(I)-(OH)-OSO_3_^−^ + OH^−^ → ≡Cu(II)-(OH) + SO_4_^•−^ + OH^−^ (3)

SO_4_^•−^ + OH^−^ → SO_4_^2−^ + •OH (4)

≡Cu(II)-OH + HSO_5_^−^ + H_2_O → O_2_**·**^−^ + 3H^+^ + SO_4_^2−^ (5)

O_2_**·**^−^ + •OH → ^1^O_2_ + OH^−^ (6)

SO_4_^•−^ + •OH+ O_2_**·**^−^ + ^1^O_2_ + CIP → Products, CO_2_, H_2_O, (7)

## 3. Materials and Methods

### 3.1. Materials and Reagents

The rice straw was taken from Mian County, Hanzhong City, Shaanxi Province. All reagents were chemically pure and purchased from Sinopharm Chemical Regent Co. Ltd. (Shanghai, China), including CuCl_2_·2H_2_O, Co (NO_3_)_2_·6H_2_O, FeSO_4_·7H_2_O, KBH_4_, ciprofloxacin, methanol (MeOH), ethanol, tert-Butanol (TBA), p-Benzoquinone (BQ), and L-histidine. Peroxymonosulfate (PMS) was purchased from Sigma-Aldrich Regent (St. Louis, MA, USA). All experiments in this work used ultrapure water.

### 3.2. Synthesis of Materials

#### Preparation of Biochar and Cu-Loaded Biochar

The preparation method of catalytic materials was described in detail. The clean and moisture-free rice straw (10 g, <1 cm) was pyrolyzed in crucible for calcination at 800 °C for 2 h with a heating rate of 10 °C S1min^−1^. The ground solids were collected under a 100-mesh sieve and marked as BC. For BC-Cu preparation, 0.2 g BC was dispersed in 50 mL 0.25 M CuCl_2_·2H_2_O aqueous solution through sonication. Then, 110 mL KBH_4_ (0.0625 M) were dropped into the above suspension for stirring for 30 min. The obtained solids were washed 5 times with ultrapure water and ethanol and dried at 60 °C for 2 h in vacuum. The syntheses of metal-decorated biochar (BC-M) were based on the above procedure; BC-Co and BC-Fe were synthesized with Co (NO_3_)_2_·6H_2_O and FeSO_4_·7H_2_O (0.25 M) under the same conditions. As comparison, a series of BC-Cu composites were obtained with the same procedure through adjusting concentrations of CuCl_2_·6H_2_O (0.13 M, 0.19 M, 0.25 M, and 0.31 M); the obtained composites were marked as BC-Cu (1:2), BC-Cu (1:3), BC-Cu (1:4), and BC-Cu (1:5).

### 3.3. Characterization Methods

The X-ray diffraction (XRD) patterns of prepared catalysts were acquired on D8AVANCE (Bruker, Ettlingen, Germany). The scanning electron microscope (SEM) images of different series materials were collected on ZEISS Sigma 300. Transmission electron microscopy (TEM) was taken on JEOL JEM-F200 to observe the microstructure of the products. The Fourier transform infrared spectra (FT-IR) of composites were obtained on VERTRX70 (Bruker, Germany). The Raman spectra of samples were measured on Horiba LabRAM HR Evolution Raman microscope (HORIBA, Kyoto, Japan). The X-ray photoelectron spectra (XPS) of the samples were characterized by Thermo Scientific K-Alpha (Waltham, MA, USA). The binding energy of survey spectra and high-resolution spectra of elements in catalysts were calibrated with specific B.E. of C 1s (248.6 eV). Electron spin resonance (ESR) signals were collected using a USA Bruker A300 spectrometer.

### 3.4. Degradation Procedure

All degradation experiments were conducted in a 250 mL beaker at 25 ± 1 °C. An amount of 20 mg of catalyst was added in 50 mL CIP solution (20 mg/L). Before addition of PMS, the suspension was stirred in the dark for half an hour to achieve adsorption-desorption equilibrium. Then, a specific amount of PMS (KHSO_5_·0.5KHSO_4_·0.5K_2_SO_4_) stock solutions were injected in suspension. At a specified time, 2 mL suspend was filtered with a 0.22 μm filter membrane.

### 3.5. Analysis Methods

The concentration of CIP was detected with high-performance liquid chromatography (UHPLC, Thermo Fisher, Ultimate 3000) equipped with a C18 column and a UV detector (273nm). The mobile phases of CIP were acetonitrile and 0.1% formic acid, and the corresponding volume ratio was 20:80. The CIP adsorption tests of BC were carried out under the same conditions without the addition of PMS.

## 4. Conclusions

In summary, iron-, cobalt-, and copper-decorated biochar was prepared using in situ chemical deposition at room temperature. Compared with the pure BC toward CIP removal, BC-Co, BC-Fe, and BC-Cu exhibited improved catalytic performance in terms of the catalytic activation of metal introduction. Among them, BC-Cu displayed satisfied performance enhancement for CIP degradation: specifically, 99.99% of the CIP in an aqueous solution over a BC-Cu (1:4)/PMS system in 40 min. The optimal BC-Cu (1:4) composite displayed excellent performance of CIP removal. In addition, BC-Cu (1:4) had superior acid-base adaptability (3.98~11.95) and anti-anion interference ability. Considering material properties and reactive oxygen species in the degradation process, an enhanced non-radical pathway with copper oxides decorated on biochar played the crucial role in CIP degradation.

## Data Availability

The data presented in this study are available in this article.

## Supplementary Materials

The following supporting information can be downloaded at: https://www.mdpi.com/article/10.3390/molecules28248094/s1, Figure S1: XRD pattern of rice straw-derived biochar; Figure S2: XRD patterns of BC-Co and BC-Fe; Figure S3: SEM image (a) and element distribution mapping (b–d) of BC-Cu (1:4). EDS spectrum (f) and content table of elements in BC-Cu (1:4).
