# Supplementary material for: Efficient Degradation of Ciprofloxacin in Water over Copper-Loaded Biochar Using an Enhanced Non-Radical Pathway
Source: Molecules. 2023 Dec 14;28(24):8094. doi: 10.3390/molecules28248094 (PMC10745511; doi:10.3390/molecules28248094)
Supplement: Supplementary file 1 [file molecules-28-08094-s001.zip › molecules-2709469-supplementary.pdf]

Supplementary Materials

Efficient Degradation of Ciprofloxacin in Water over Copper Loaded Biochar by Enhanced Non-Radical Pathway

*Guo Ting *, Yang Qinyu, Qiu Ruoqi, Gao Jie, Shi Jingzhuan, Lei Xiaoyun and Zhao Zuoping **

School of Chemistry and Environmental Science, Shaanxi University of Technology, Hanzhong 723001, China

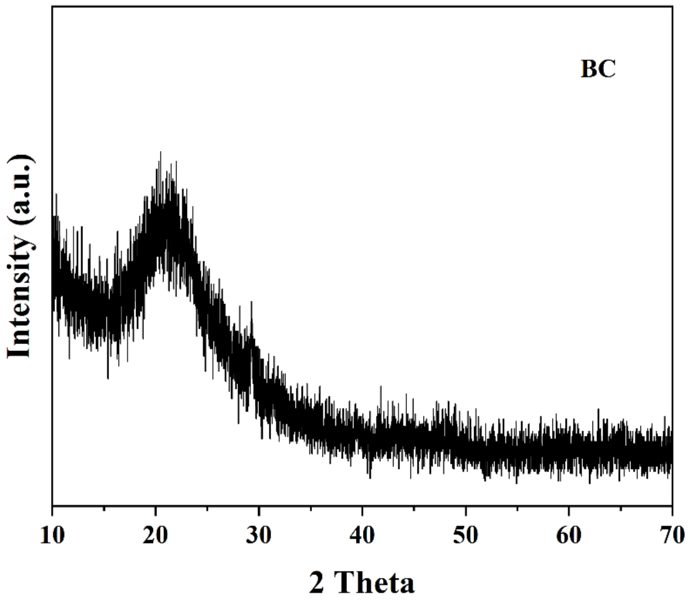

Figure S1. XRD pattern of rice straw derived biochar.

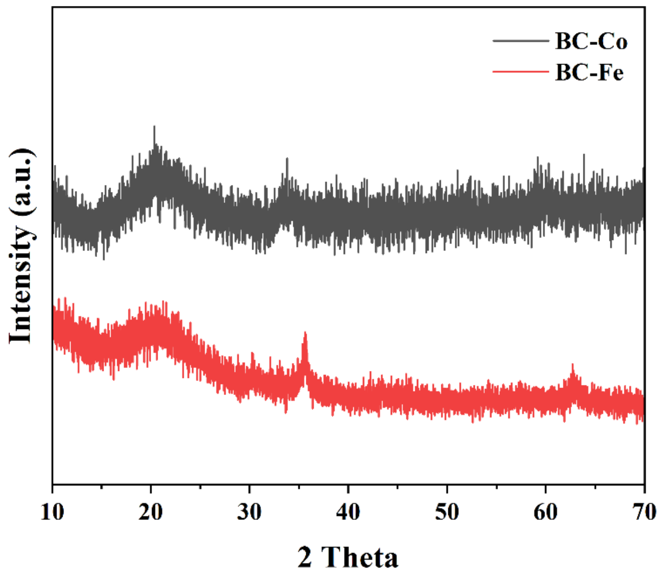

Figure S2. XRD patterns of BC-Co and BC-Fe.

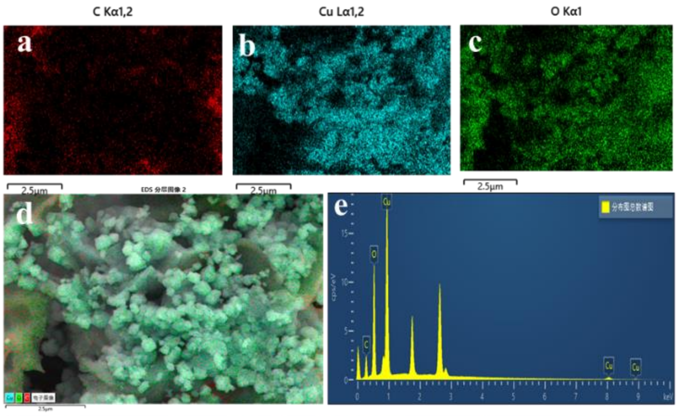

Element	wt%	Wt % Sigma	At%
C	16.92	0.29	36.05
O	25.47	0.19	40.75
Cu	57.61	0.27	23.21
Total	100.00		100.00

Figure S3. Elemental mapping (**a-d**) of C, Cu, O in BC-Cu (1:4). EDS spectrum (**e**) of BC-Cu (1:4). Element content table of BC-Cu (1:4).

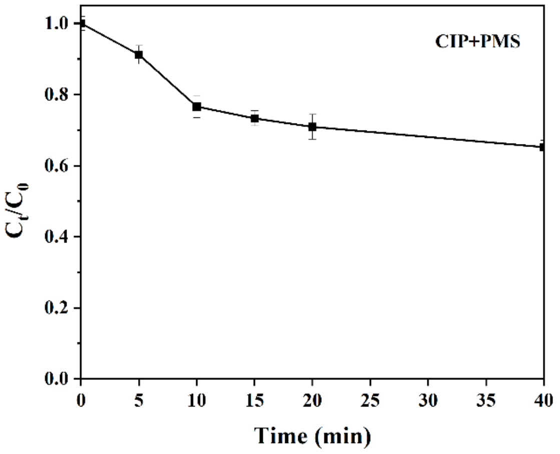

Figure S4. CIP degradation by PMS. CIP concentration: 20 mg·L^{-1}, initial pH: 6.80, PMS concentration: 0.20 g·L^{-1}.
